# Identification of two novel linear epitopes on the E165R protein of African swine fever virus recognized by monoclonal antibodies

**DOI:** 10.3389/fvets.2024.1392350

**Published:** 2024-08-06

**Authors:** Jian He, Jieqiong Li, Mingzhan Luo, Yangkun Liu, Jingchen Sun, Lunguang Yao

**Affiliations:** ^1^Henan Provincial Engineering and Technology Center of Health Products for Livestock and Poultry, Henan Field Observation and Research Station of Headwork Wetland Ecosystem of The Central Route of South-to-North Water Diversion Project, School of Life Science and Agricultural Engineering, Nanyang Normal University, Nanyang, China; ^2^Guangdong Provincial Key Laboratory of Agro-animal Genomics and Molecular Breeding, College of Animal Science, South China Agricultural University, Guangzhou, China

**Keywords:** African swine fever, African swine fever virus, E165R protein, monoclonal antibody, conserved epitope

## Abstract

African swine fever (ASF) is a highly fatal infectious disease in pigs, caused by the African swine fever virus (ASFV). It is characterized by short disease duration and high morbidity and mortality. In August 2018, ASF was first reported in China and it subsequently spread rapidly throughout the country, causing serious economic losses for the Chinese pig industry. Early detection plays a critical role in preventing and controlling ASF because there is currently no effective vaccine or targeted therapeutic medication available. Additionally, identifying conserved protective antigenic epitopes of ASFV is essential for the development of diagnostic reagents. The E165R protein, which is highly expressed in the early stages of ASFV infection, can serve as an important indicator for early detection. In this study, we successfully obtained high purity soluble prokaryotic expression of the E165R protein. We then utilized the purified recombinant E165R protein for immunization in mice to prepare monoclonal antibodies (mAbs) using the hybridoma fusion technique. After three subclonal screens, we successfully obtained three mAbs against ASFV E165R protein in cells named 1B7, 1B8, and 10B8. Through immunofluorescence assay (IFA) and Western blot, we confirmed that the prepared mAbs specifically recognize the baculovirus-expressed E165R protein. By using overlapping truncated E165R protein and overlapping peptide scanning analysis, we tentatively identified two novel linear B cell epitopes (^13^EAEAYYPPSV^22^ and ^55^VACEHMGKKC^64^) that are highly conserved in genotype I and genotype II of ASFV. Thus, as a detection antibody, it has the capability to detect ASFV across a wide range of genotypes, providing valuable information for the development of related immunodiagnostic reagents.

## 1 Introduction

African swine fever (ASF) is a severe and acute infectious disease of pigs caused by the African swine fever virus (ASFV) (1). The acute phase of ASF can cause 100% mortality in pigs, and symptoms include lethargy, bloody diarrhea, hyperthermia, and vascular changes (2). ASFV transmission primarily occurs through direct contact between infected and healthy pigs. ASF can be transmitted orally and nasally, but also through soft tick bites, scratches, or injections (3, 4). Due to the complex structure and diverse functionality of ASFV, controlling the spread of the virus presents challenges. With the absence of an available commercial vaccine, culling measures are necessary to contain the epidemic (5). Therefore, the development of highly sensitive and specific diagnostic methods becomes crucial in promptly detecting and isolating ASFV-infected pigs.

ASF has had devastating economic and ecological consequences worldwide and is considered one of the most destructive diseases (6). It first emerged in Kenya in the 1920s and re-emerged in Georgia in 2007, rapidly spreading across sub-Saharan Africa, the Caribbean, and Eastern Europe, until it reached China in 2018. Since then, it has continued to spread to other Asian countries (6–8). One of the characteristics that distinguish ASFV from different strains or generations is the duplication and deletion of specific sequences in its genome. The C-terminus of the protein p72 encoded by the B646L gene has been subjected to phylogenetic analysis, revealing that ASFV is currently classified into 24 genotypes (genotype I to XXIV). Notably, the highly virulent genotype II strain is the main strain currently transmitted in China (1, 9).

ASFV is a DNA virus, 260–300 nm in diameter, with a genome of 170–190 kb in length. It encodes more than 170 proteins, including enzymes, structural proteins, and scaffolding proteins (10–12). The replication of ASFV primarily occurs within macrophages and monocytes. The viral infection undergoes attachment, internalization, genome proliferation, viral assembly, and release (13, 14). Ultimately, the virus is released from cells as an outgrowth (15). The dUTPase (E165R), K196R, and A240L proteins encoded by the *E165R*, *K196R*, and *A240L* genes, are the major nucleotide metabolizing enzymes responsible for providing energy during viral replication (16). The dUTPase has been shown to be associated with innate immunity (17, 18) and can activate the nuclear factor-kappa B (NF-κB) signaling pathway (19). The ASFV *E165R* gene encodes a deoxyuridine triphosphate nucleotidohydrolase (20). Similar to other dUTPase enzymes, the dUTPase of ASFV has a trimeric structure, and it is expressed throughout infection, playing a crucial role in maintaining the integrity of viral replication. ASFV has been found to encode both a base excision repair system (BER) and a dUTPase. The BER system repairs DNA base damage caused by hydrolysis or oxidation, while the dUTPase degrades 2′-deoxyuridine 5′-triphosphate (dUTPase) in the cytoplasm, reducing the likelihood of uracil incorporation in viral DNA replication errors and contributing to high fidelity viral genome replication (21). Deletion of the E165R gene strongly inhibits ASFV replication *in vitro*, indicating its correlation with virulence and replication efficiency (21). However, recent studies have shown that deletion of the *dUTPase* gene does not affect ASFV replication and virulence (22). Monoclonal antibodies (mAbs) that target the highly conserved region of E165R can specifically inhibit the dUTPase activity of E165R (23). Additionally, ASFV dUTPase exhibits remarkable tolerance to high temperatures, up to 83°C, making it a promising enzymatic tool (24). Epitopes, which are the basic structural and functional units of antigenic proteins, play a crucial role in disease diagnosis, vaccine design, and other applications that require specific immune responses.

In this study, E165R was chosen as the target protein, and three monoclonal antibodies (mAbs) were successfully generated against the prokaryotic expression of the E165R protein. By performing overlapping truncated E165R protein and peptide scanning analysis, two novel conserved epitopes targeted by the mAbs were identified. In conclusion, these findings lay the foundation for the development of new diagnostic reagents.

## 2 Materials and methods

### 2.1 Genes, cells, and animals

The recombinant plasmid pET32a-E165R of ASFV Pig/HLJ/2018 strain (Genbank: MK333180.1), was synthesized by Sangon Bioengineering Co., Ltd. (Shanghai, China). Sf9 cells and the recombinant baculovirus Ac MutliBac-E165R (the *E165R* gene driven by polyhedrin promoter) were stored in our laboratory. SP2/0 cells were donated by the Key Laboratory of Animal Immunology of Henan Academy of Agricultural Sciences. Six-week-old female BALB/c mice were obtained from Autobio Diagnostics Co., Ltd. (Zhengzhou, China).

### 2.2 Preparation and identification of the recombinant protein E165R

The recombinant plasmid pET32a-E165R was transformed into *E. coli* Bl21 (DE3) competent cells (Weidi, Shanghai, China). After screening for ampicillin resistance, positive monoclonal bacteria were selected for expression induction. Specifically, Isopropyl-β-D-thiogalactopyranoside (IPTG) inducer was added at a final concentration of 0.5 mmoL/L when the OD600 nm value of the bacterial solution reached approximately 0.4–0.6. The induction was carried out at 37°C for 6 h. The bacterial solution products were collected by centrifugation at 12,000 g and then washed three times with 1 × PBS (Solarbio, Beijing, China). Following sonication and lysis of the bacteria, the supernatants were isolated and their solubility was analyzed using SDS-PAGE. The protein was purified using a Ni-NTA resin-based column (Solarbio, Beijing, China) according to the instructions. Elution of the target protein was achieved by varying the concentration of imidazole in the elution solution. The purified sample of E165R protein was analyzed by SDS-PAGE and Western blot using His-tag mAb (Proteintech, Wuhan, China).

### 2.3 Animal experiment and preparation of E165R mAbs

All animal protocols were conducted with the ethics committee guidelines of Nanyang Normal University. Six-week-old BALB/C mice were immunized with purified E165R protein. For the primary immunization, a 20 μg dose of the protein was mixed with an equal amount of complete Freund’s adjuvant. Booster immunizations were subsequently administered on days 14 and 28. Seven days after the final immunization, blood samples were collected from the tail veins to determine serum antibody titers using indirect ELISA. The mouse with the highest serum titer was selected for subsequent intraperitoneal booster immunizations. Positive serum samples were collected from the eye blood after three days, and splenocytes were isolated from mice with high serum potency. These splenocytes were then fused with SP2/0 myeloma cells in logarithmic growth using PEG 1500 treatment. Cells were screened, cultured in an HAT-containing medium, and cultured in 96-well plates. Positive hybridoma cells were selected 10 days after fusion, and three subclonal screenings were performed using the limited dilution method to obtain stably cultured mAbs. The monoclonal cell lines were then amplified through *in vivo* induction of ascites.

### 2.4 Indirect ELISA

Indirect ELISA method was utilized to detect the titer of anti-E165R mAb ascites. The E165R protein was diluted to a concentration of 1 μg/mL using 1 × ELISA coating buffer (Solarbio, Beijing, China). Then, 100 μL of the diluted solution was added per well onto the ELISA plate. The ELISA plate was coated with 5% skim milk powder and incubated overnight. The mAb ascites were diluted in a double ratio dilution from 1:400 to 1:819,200 as the primary antibody. The secondary antibody, HRP-conjugated goat anti-mouse IgG (H + L) (diluted 1:5,000, Proteintech, Wuhan, China), was then added and incubated in the wells for 1 h at 37°C. Next, 100 μL of TMB was added to the reaction and incubated for 10 min. The reaction was then stopped by adding 2 moL/L H_2_SO_4_, and the optical density at 450 nm was measured by reading the plate.

According to the instructions provided by the mouse monoclonal antibody isotype identification kit (Proteintech, Wuhan, China), mAbs isotypes were identified by reading the OD450 nm value.

### 2.5 Immunofluorescence assay

The specificity of the mAb was further confirmed using the IFA method. Sf9 cells were infected using the Ac MutliBac-E165R recombinant baculovirus, while uninfected served as a negative control (NC). 72 h after infection, the supernatant was removed, and the cells were fixed in 4% paraformaldehyde at room temperature (RT) for 20 min. Then, the cells were permeabilized with 0.1% Triton X-100, rinsed three times with PBST, and blocked with 5% skim milk powder at 37°C for 1 h. Subsequently, the cells were incubated with the goat anti-mouse IgG H&L (FITC) (diluted 1:100, Abcam, UK) at 37°C for 1 h. After washing, the cell nuclei were stained with DAPI (Solarbio, Beijing, China), and the cells were observed under an inverted fluorescence microscope.

### 2.6 Western blot

Sf9 cells were infected with Ac MutliBac-E165R recombinant baculovirus, while uninfected cells were established as negative control. After 48 h of infection, the cell samples were collected, and 50 μg sample were used then analyzed by Western blotting with E165R mAbs as primary antibodies. PVDF membranes were coated with 5% skim milk powder at 37°C for 1 h. Subsequently, PVDF membranes were incubated with the anti-ASFV E165R mAbs (diluted 1:2,000) at RT for 1 h. After three washes with PBST, then incubated with the HRP-conjugated goat anti-mouse IgG (diluted 1:5,000) at RT for 1 h. The membranes were washed three times and then subjected to enhanced chemiluminescence (ECL) color development in the dark.

### 2.7 Design and synthesis of polypeptides

To determine the epitopes recognized by the mAb. The E165R protein was truncated into four segments containing regions of overlap, and ligated to the prokaryotic expression vector pET32a (+) for induced expression, named A, B, C, and D, respectively (primer used shown in Table 1). Subsequently, the epitopes recognized by mAb were identified by western blot analysis. Based on the western blot results, 12 peptide fragments containing six overlapping amino acids were synthesized. To facilitate the coupling to the BSA protein, a C-cysteine (C) was added at the N-terminal end of the peptide fragment to ensure the presence of at least one cysteine residue. All peptides were synthesized by GenScript (Nanjing, China) (purity >95%).

**Table 1:** Primer used in this study.

Primer names	Sequences	Fragments
A	F:CGCGGATCCATGGCAACAAATTTTTTTG R:CCGAATTCGTGGTGGTGGTGGTGGTGTTTTTTGCCCATGTGTTC	1–63 aa
B	F:CGCGGATCCATGGCAACAAATTTTTTTG R:CCGAATTCGTGGTGGTGGTGGTGGTGGTTTGCAAGGATGAGCAG	1–85 aa
C	F:CGCGGATCCATGGCAACAAATTTTTTTG R:CCGAATTCGTGGTGGTGGTGGTGGTGTTCTTTGGCCCATATTTG	1–116 aa
D	F:CGCGGATCCATGGAACACATGGGCAAAG R:CCGAATTCGTGGTGGTGGTGGTGGTGAGTTCTCATAATCCCGGC	58–165 aa

### 2.8 Identification and screening of polypeptides

The antigenic epitopes recognized by the mAbs were identified by the dot-blot method. The peptide was conjugated to the BSA carrier protein using Sulfo-SMCC and the coupled peptide was spotted onto a nitrocellulose filter membrane (NC). A negative control (BSA protein) and a positive control (E165R protein) were included. The prepared mAbs were used as the primary antibody, and HRP-conjugated goat anti-mouse IgG (H + L) was used as the secondary antibody. The reactivity of the mAbs with the peptide was detected using an ECL reagent.

Peptide-ELISA assay for localization of mAbs. Peptides coupled with BSA were to a concentration of 5 μg/mL using ELISA coating buffer, followed by 100 μL/well coated in ELISA plates with BSA negative control and E165R protein positive control. The plates were incubated at 4°C overnight. Three replications were performed for each experimental group. After incubation, 100 μL of the diluted mAbs (diluted 1:1,000) was added as the primary antibody, followed by the addition of HRP-conjugated goat anti-mouse IgG (diluted 1:3,000) as the secondary antibody. Finally, the color development reaction was carried out using 100 μL of TMB per well, followed by the addition of 50 μL of 2 M H_2_SO_4_ to terminate the reaction.

### 2.9 Homology analysis

Homology analysis of the E165R mAb recognition epitope in this study was performed using MEGA 11 (Mega Limited, Auckland, New Zealand). The E165R sequences of 35 ASFV strains from different regions were compared individually. The ASFV E165R structure (PDB: 6LIS) was simulated using PyMOL, and the identified epitopes were labeled.

## 3 Results

### 3.1 Expression and purification of E165 protein

**Figure 1:**
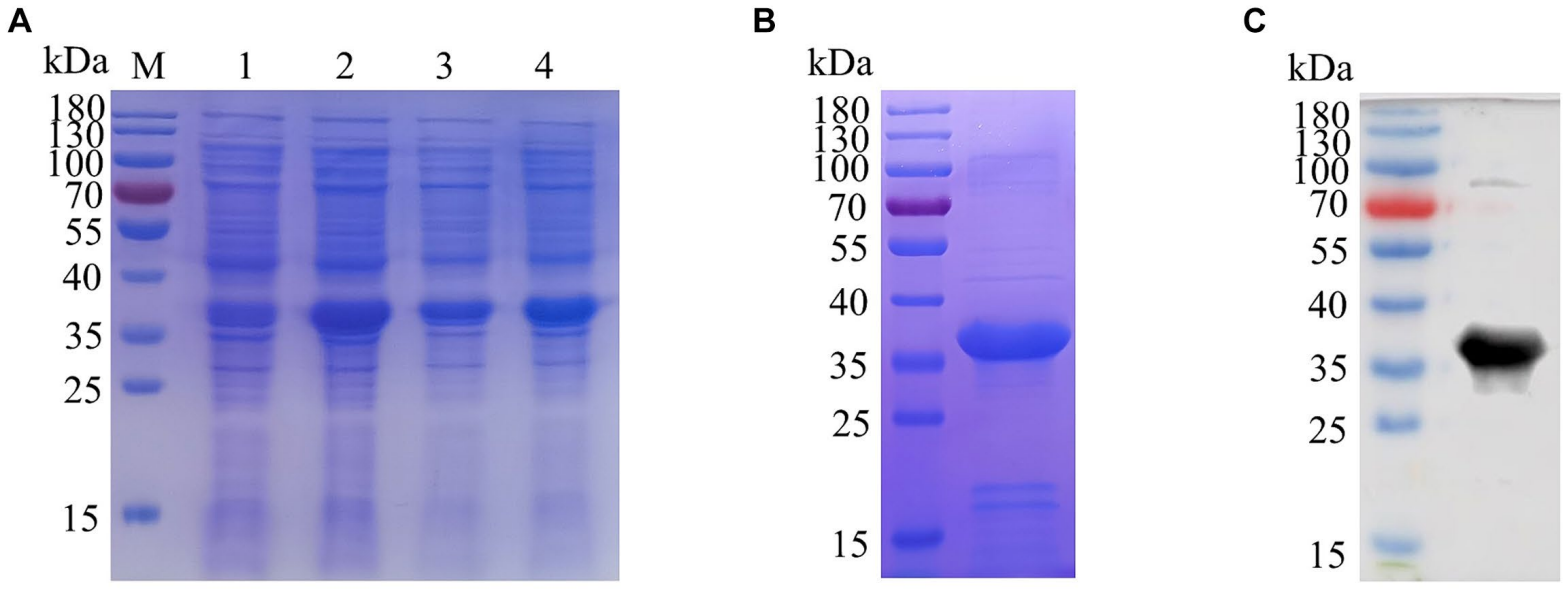
Expression and purification of the E165R recombinant protein in the *E. coli* prokaryotic expression system. **(A)** SDS gel of E165R recombinant protein expression; M: Prestained protein ladder; lane 1: Cell proteins of pET32a after inducement; lane 2: Cell proteins of pET32a-E165R after inducement; lane 3: Supernatant of lysed pET32a-E165R after inducement; lane 4: Precipitation of lysed pET32a-E165R after inducement. **(B)** SDS-PAGE analysis of purified E165R protein. **(C)** Western blot analysis of purified E165R protein.

The recombinant plasmid pET32a-E165R was transformed into *E. coli* BL21(DE3) for expression through induction with IPTG. Results showed that the results showed high-level expression of the E165R protein predominantly in a soluble form, with a molecular mass of approximately 38 kDa (Figure 1A). Following expression, the recombinant E165R protein was purified using Ni Sepharose 6FF, resulting in a purity exceeding 90% (Figure 1B). Subsequently, the identity of the recombinant E165R protein was confirmed by Western blot, which clearly demonstrated its specific interaction with the His tag mAbs (Figure 1C).

### 3.2 Preparation of mAbs against E165R protein

**Figure 2:**
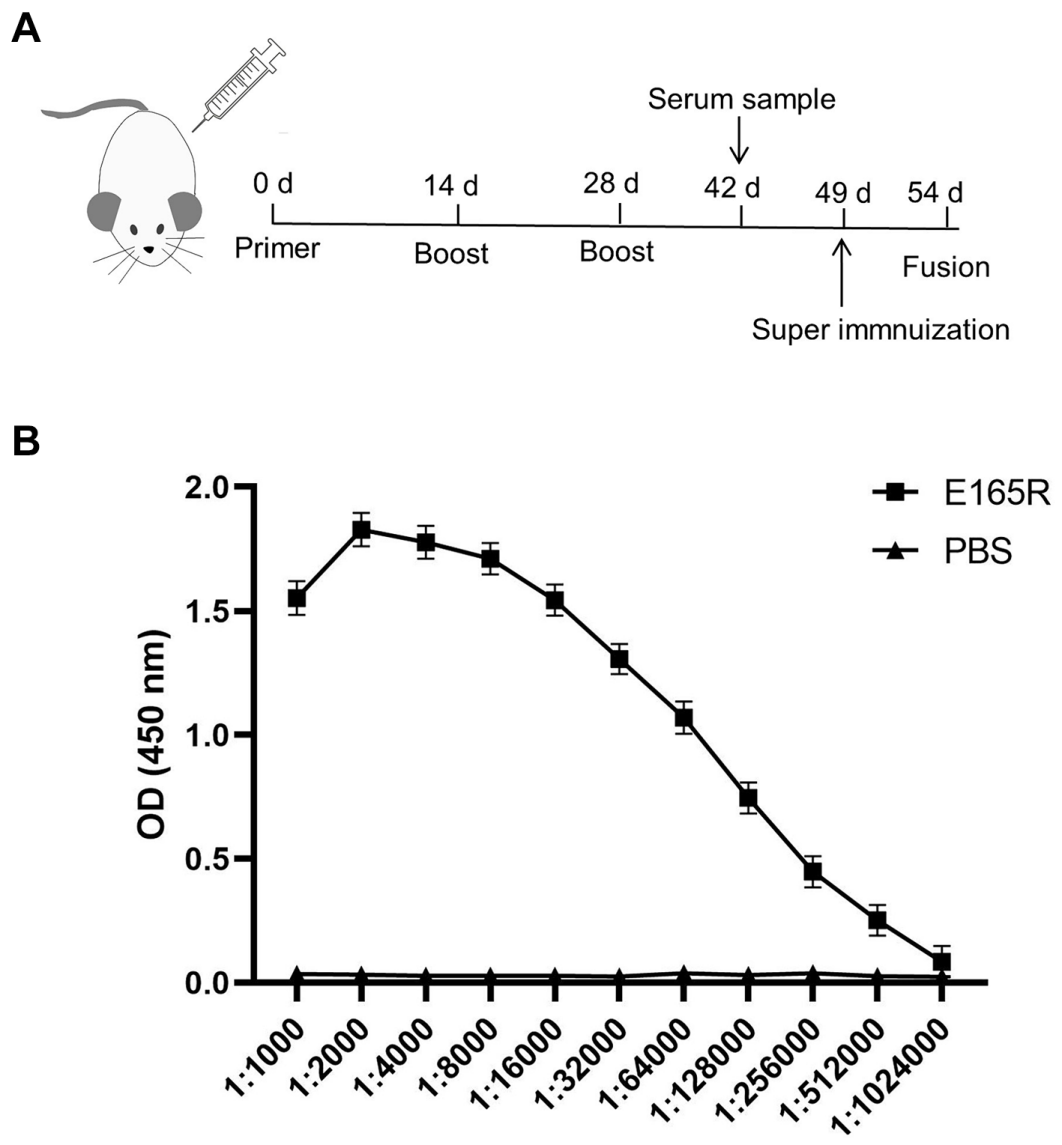
Determination of the anti-serum titer in serum before fusion **(A)** Strategy development of immune antigenic proteins in mice. **(B)** The purified E165R protein was diluted to 1 μg/mL and then coated in the microplate plate, and the mouse antibody titer was determined by indirect ELISA.

BALB/c mice were immunized with purified E165R protein. After the immunization procedure (Figure 2A), tail vein blood was collected on the 7th day after the third immunization. Serum antibody potency was measured using ELISA, results showed that the antibody titer of mouse serum was 1:5.12 × 10^5^, demonstrating the successful production of specific antibodies against ASFV E165R protein (Figure 2B). Three stable growth-targeted E165R protein mAb hybridoma cell lines, named 1B7, 1B8, and 10B8, were obtained through limited dilution subcloning and ELISA methods.

### 3.3 Three mAbs exhibited strong reactivity with recombinant E165R protein

**Figure 3:**
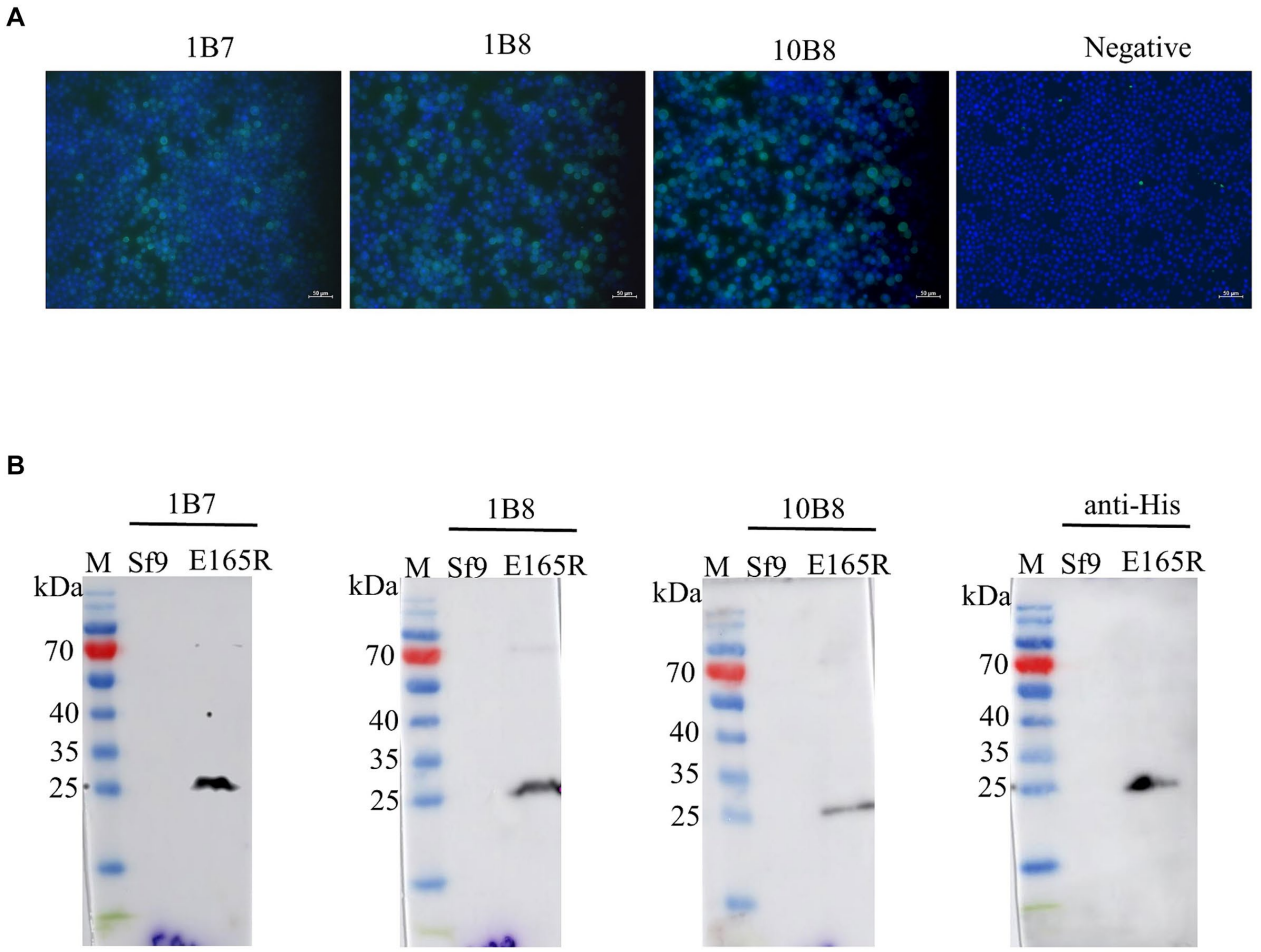
Three mAbs reacted well with the Ac MutliBac-E165R recombinant baculovirus **(A)** The reactivity of mAbs was analyzed with IFA. IFA analysis of the reactivity of the mAbs. Green was the reactions of mAbs 1B7, 1B8, and 10B8 with recombinant baculovirus Ac MultiBac-E165R; the blue color is Sf9 cell nuclei after DAPI staining, indicating that the prepared mAbs can react with the recombinant baculovirus E165R. **(B)** Western blot analysis of the reactivity between E165R protein and mAbs. Cells uninfected with recombinant baculovirus E165R were used as a negative control.

The specificity of the mAbs was evaluated by infecting Sf9 cells with the recombinant baculovirus Ac MultiBac-E165R using Western blot analysis and IFA. The IFA results demonstrated specific green fluorescence, indicating that the generated mAbs specifically recognized the E165R protein expressed in Sf9 cells (Figure 3A). Furthermore, the results in Figure 3B confirmed that all three mAbs were capable of recognizing the E165R proteins.

### 3.4 Identification of potency and subtype of three mAbs

**Figure 4:**
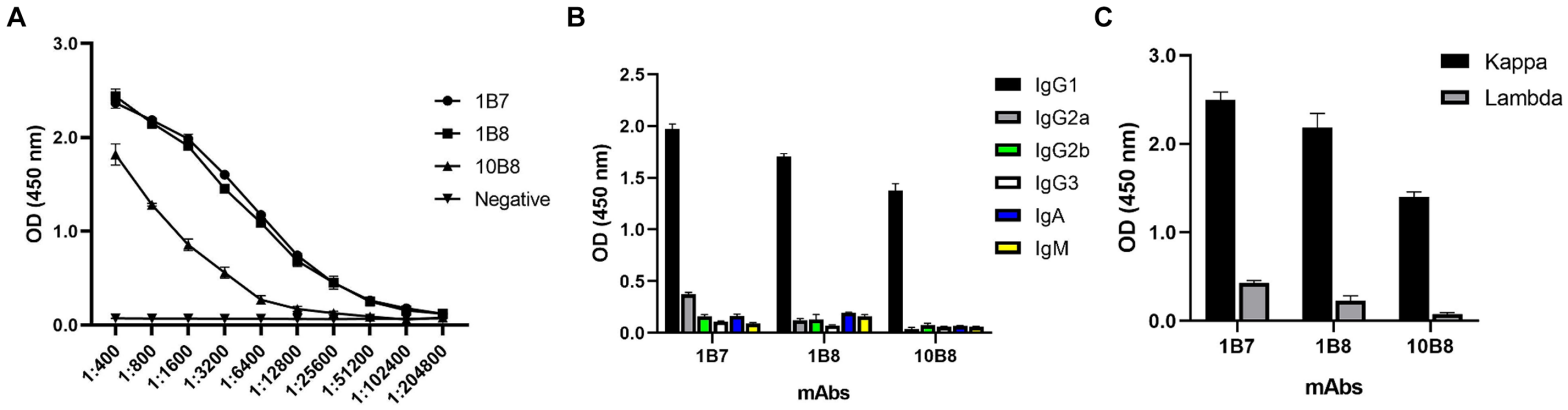
Ascites titers and subtype identification of three mAbs against E165R protein. **(A)** Ascites titers of E165R mAbs performed by ELISA. **(B)** Heavy chains of mAbs; **(C)** Light chains of mAbs.

To further reveal the characteristics of the three mAbs, the potency of the mAbs was determined by indirect ELISA. A large number of mAbs were prepared by induction of ascites and the titer was measured as follows: 1B7 (1:1.024 × 10^5^), 1B8 (1:1.024 × 10^5^), and 10B8 (1:1.28 × 10^4^) (Figure 4A). Furthermore, a commercial kit was utilized to confirm that all prepared mAbs belonged to the IgG1 subtype (Figure 4B) and contained the kappa light chain (Figure 4C).

### 3.5 Epitope mapping of the three mAbs

**Figure 5:**
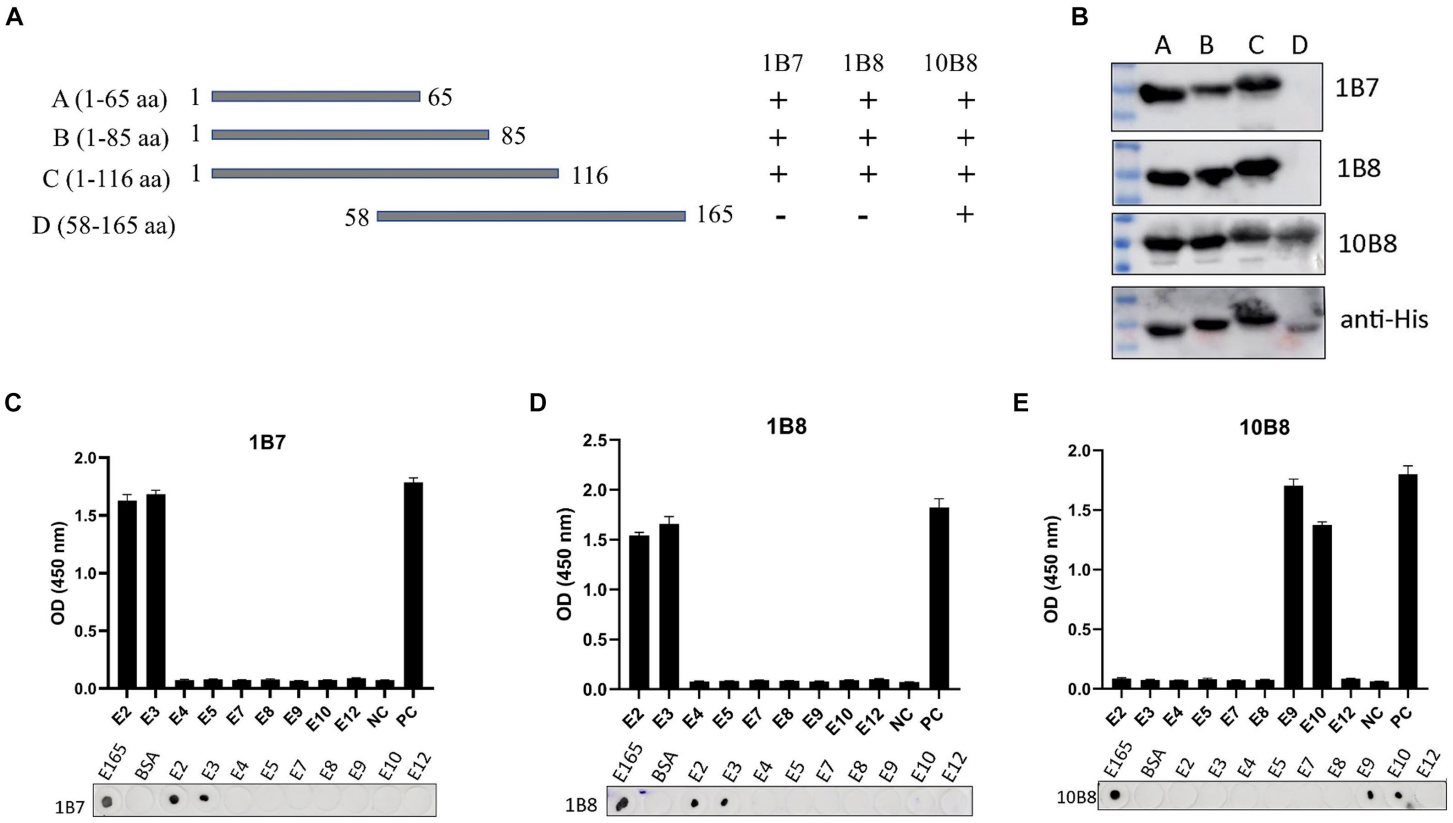
Mapping epitopes of three mAbs against the E165R protein. **(A)** Design options for the preliminary screening of epitopes. **(B)** A series of E165R truncated fragments were constructed into pET32a (+) and successfully expressed in *E. coli* BL21 (DE3) competent cells. Prepared mAbs or anti-His mAb were used as primary antibodies, respectively, and the truncated E165R was detected by Western blot. **(C)** Reactions of anti-E165R mAbs with 9 short peptides were detected by dot-blot and peptide-ELISA **(C–E)**. BSA protein was the negative control and E165R protein was the positive control. Each experiment was carried out three times, and the error bars represent the standard deviation (SD).

Epitope mapping of three mAbs was determined using Western blot, dot-blot, and peptide-ELISA. First, we designed and expressed four overlapping truncated E165R peptides (Figure 5A). To ensure detection, the truncated fragments were expressed using the pET32a vector, which incorporates an 11.8 kD trxA tag sequence at the N-terminus. Western blot analysis demonstrated that proteins A, B, and C were recognized by 1B7 and 1B8, whereas proteins A, B, C, and D were recognized by 10B8. These findings indicated that the regions recognized by 1B7, 1B8 and were between 1–58 aa, while the region recognized by 10B8 was between 58–65 aa (Figure 5B).

Subsequently, we designed twelve short peptides, each containing 6 overlapping amino acids (Table 2). However, only nine of these peptides were successfully synthesized due to their hydrophobic properties. It was further validated by dot-blot and peptide-ELISA. In particular, peptides E2 and E3 were recognized by 1B7 and 1B8 (Figures 5C,D), and peptides E9 and E10 were recognized by 10B8 (Figure 5E). As a result, it was determined that the recognition sites of 1B7 and 1B8 were ^13^EAEAYYPPSV^22^, and 10B8 was ^55^VACEHMGKKC^64^.

**Table 2:** Design of overlapped peptides cover the E165R protein in this research.

Peptides	Sequences	Peptides	Sequences
E1	CMATNFFIQPITEEAEA	E7	CSDLVLQPGLNIVRLH
E2	CIQPITEEAEAYYPPSV	E8	QPGLNIVRLHIKVACE
E3	CEAEAYYPPSVITNKRK	E9	VRLHIKVACEHMGKKC
E4	CPPSVITNKRKDLGVDV	E10	VACEHMGKKCGFKIMA
E5	NKRKDLGVDVYCCSDL	E11	GKKCGFKIMARSSMCT
E6	GVDVYCCSDLVLQPGL	E12	KIMARSSMCTHERLLI

### 3.6 Conservative analysis of mAb epitopes

**Figure 6:**
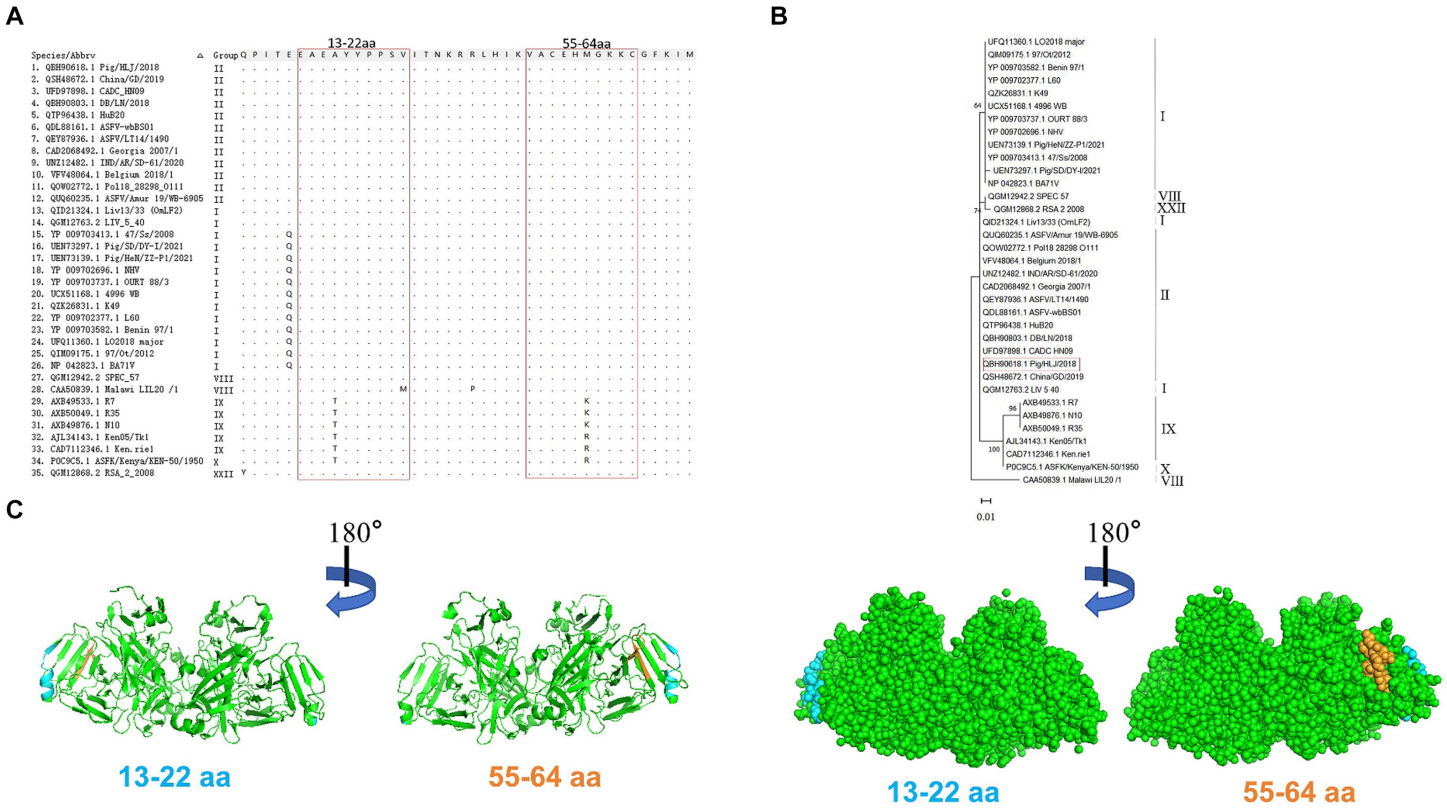
Conservative analysis of epitopes. **(A)** Amino acid sequences of E165R in 35 ASFV strains of different genotypes were aligned. The amino acid coordinates of each sequence are at the top and left of the sequence, and matching residues are indicated by black dots. Boxed in red are the epitopes ^13^EAEAYYPPSV^22^ and ^55^VACEHMGKKC^64^. **(B)** ASFV E165R genetic evolution analysis [maximum likelihood method (ML)]. **(C)** The prediction region of the epitope ^13^EAEAYYPPSV^22^ (blue color) and the epitope ^55^VACEHMGKKC^64^ (orange color) are located on the spatial structure of the ASFV E165R protein (PDB: 6LIS).

Alignment analysis was performed to evaluate the conservation of the epitopes identified among 35 ASFV isolates belonging to six genotypes. Results of amino acid alignment shown in Figure 6A, the epitopes of ^13^EAEAYYPPSV^22^ were highly conserved in ASFV genotypes I, II, and XXII. Conversely, the epitopes ^55^VACEHMGKKC^64^ exhibited high conservation in genotypes I, II, VIII, and XXII. Phylogenetic tree analysis confirmed that the E165R sequence used in this study has a close similarity to the genotype I and II epidemic strain (Figure 6B). As genotypes I and II are the main ASFV epidemic strains in Asia and Europe, the conserved epitopes revealed in this study offer a practical strategy and option for developing ASFV detection methods.

PyMOL software was used to simulate the E165R protein structure and to observe the spatial distribution of the epitopes identified by the mAbs developed in this study (Figure 6C).

## 4 Discussion

Acute ASF has a lethality rate of up to 100% and has a devastating impact on the pig farming industry. China, as the main breeding country for pigs in the world, has caused huge economic losses due to the ASF (25). However, the lack of sufficient knowledge about the mechanisms of host and ASFV immune responses has hindered vaccine development (26, 27). The complexity of ASFV itself and the limited understanding of virulence factors in different strains, as well as their associated protective genes, have created further challenges in the treatment and vaccine development processes. As a result, safe and effective preventive vaccines and targeted therapeutic agents have yet to be identified (28). The emergence of novel features in ASF epidemics, including naturally attenuated and highly virulent strains, coupled with the long incubation period of infected pigs, has made ASFV detection even more difficult. Thus, there is an urgent need for the development of a rapid and sensitive diagnostic reagent for ASF.

dUTPase is an essential hydrolase found in a wide range of organisms, including prokaryotes, eukaryotes, DNA viruses, and RNA viruses (29, 30). Current research on dUTPase primarily focuses on viruses. These studies confirm that dUTPase plays a crucial role in facilitating efficient viral replication within the host organism. Moreover, dUTPase has been identified as a significant factor in determining viral virulence (31, 32). Additionally, certain monomeric dUTPases have broader implications beyond nucleotide synthesis and metabolism. For example, the dUTPase encoded by the Epstein–Barr virus (EBV) has been observed to impact the host immune system by promoting the production of pro-inflammatory cytokines and exerting non-enzymatic regulatory effects (33, 34).

The ASFV E165R gene encodes a dUTPase enzyme that primarily functions to prevent the misincorporation of uracils into viral DNA, thus ensuring the fidelity of genome replication (16, 35, 36). This enzyme catalyzes the hydrolysis of deoxyuridine triphosphate (dUTP) into dUMP and pyrophosphate, effectively removing dUTP from DNA synthesis (24). Both dTTP and dUTP can be incorporated into DNA with the same efficiency by DNA polymerase during DNA synthesis. However, high levels of dUTP lead to the misincorporation of uracil (37), which is crucial for the cell viability of all organisms (30, 37). Similar to most dUTPases, ASFV dUTPase exhibits a trimeric structure with an active enzyme center composed of distinct subunits. It is believed that the expression of this enzyme during both the early and late phases of infection plays a crucial role in maintaining the integrity of viral replication (38).

The high oxidative environment of macrophages, along with the significantly higher percentage of dUTP relative to dTTP, results in ASFV heavily relying on its encoded dUTPase (24). The dUTPase enzyme plays a crucial role in maintaining the fidelity and integrity of ASFV’s viral genome. Oliveros et al. (38) demonstrated that dUTPase influences the efficient replication of ASFV in porcine macrophages by generating ASFV dUTPase deletion mutants. Additionally, EBV dUTPase upregulates the expression of TNF-α and IL-6 via the NF-κB signaling pathway (17, 18). Treatment of cancer cells with the 2-deoxy-5 inhibitor TAS-114, in combination with 2-deoxy-5-fluorouracil (FDURD), has been shown to increase levels of FDUTP and dUTP, as well as the misincorporation of 5-FU and uracil, resulting in increased cancer cell mortality (39–41). Hence, inhibition of dUTPase enhances the antitumor activity of fluoropyrimidines. Furthermore, dUTPase is strongly correlated with ASFV’s virulence and its high level of replication efficiency (19, 42). Although dUTPase proteins of different species origins have varying molecular weights, they all contain amino acid sequences of five typically conserved motifs (motif I-V), with motif III generally considered the catalytic center of the enzyme activity of dUTPase (43).

The recombinant protein E165R, produced in this study, exhibits high expression levels and solubility, facilitating the preservation of its natural structure. Additionally, mAbs exhibit strong reactivity toward the heterologously expressed E165R protein. Subsequently, mAb epitopes were recognized using overlapping truncated E165R proteins in combination with peptide scan analysis. Unfortunately, the mAbs do not achieve complete neutralization virus activity. In this study, two novel conserved antigenic epitopes were screened: ^13^EAEAYYPPSV^22^ and ^55^VACEHMGKKC^64^. The ^55^VACEHMGKKC^64^ epitope is located in the conserved Motif I region of the dUTPase enzyme. Since E165R is highly expressed in the early stages of ASFV infection, antibodies targeting this conserved region are important for early virus detection. Analyzing the sequence conservation of this region, the ^13^EAEAYYPPSV^22^ epitope, recognized by mAbs 1B7 and 1B8, is highly conserved in ASFV genotypes I, II, and XXII, making it a valuable detection antibody with broad applicability. Similarly, the ^55^VACEHMGKKC^64^ region recognized by mAb 10B8 is highly conserved in genotypes I, II, VIII, and XXIII, and 1–2 base difference in genotypes IX and X. Consequently, the epitope information obtained in this study holds significant implications for future ASF diagnosis.

In conclusion, we obtained high levels of expression and high solubility of the E165R protein by utilizing the *E. coli* prokaryotic expression system. Furthermore, we screened three strains of mAbs that specifically target ASFV E165R, resulting in the identification of two novel conserved epitopes. These findings offer valuable theoretical groundwork for the future development of diagnostic reagents.

## Data availability statement

The raw data supporting the conclusions of this article will be made available by the authors, without undue reservation.

## Ethics statement

The animal study was approved by Animal Care Committee of Nanyang Normal University, China. The study was conducted in accordance with the local legislation and institutional requirements.

## Author contributions

JH: Conceptualization, Data curation, Formal analysis, Methodology, Writing – original draft, Writing – review & editing. JL: Conceptualization, Data curation, Formal analysis, Methodology, Writing – original draft. ML: Formal analysis, Methodology, Writing – original draft. YL: Data curation, Supervision, Writing – original draft. JS: Conceptualization, Supervision, Writing – review & editing. LY: Conceptualization, Funding acquisition, Resources, Supervision, Writing – review & editing.
